# Central Neurocytoma with Hemorrhagic Presentation Case Report and Review of the Literature

**DOI:** 10.1155/2022/9731987

**Published:** 2022-03-10

**Authors:** Mohammad Nasser Alsadiq, Zhra Muneer Al Sadah, Sohail Butt, Anwar Ali Aldahmen

**Affiliations:** ^1^Department of Neurosurgery, Dammam Medical Complex, Dammam, Saudi Arabia; ^2^College of Medicine, Imam Abdulrahman Bin Faisl University, Dammam, Saudi Arabia; ^3^Department of Pathology Dammam Regional Laboratory, Saudi Arabia

## Abstract

Central neurocytoma (CN) is slow-growing rare intraventricular tumor that represents 0.25-0.5% of all intracranial tumors in adults. Typically, it is seen in young adults, yet with surgical resection, it has excellent prognosis. Due to CN rarity and its feature similarities with other common tumors, misdiagnosis can be an issue. With no pathognomonic clinical features of CN, a proper diagnosis can be achieved by radiological imaging, magnetic resonance spectroscopy, histopathology assessment, and immunohistochemistry. Therefore, this is a case report of a 17-year-old male who presented with right lateral ventricular CN with intraventricular hemorrhage. Subtotal tumor resection was carried out via right transcortical approach. Subtle improvement for the patient condition was noted.

## 1. Introduction

Central neurocytoma (CN) is a benign tumor with an intraepithelial location arising from germinal matrix cells in the septum pellucidum (near foramina of Monro) or the periventricular region. It is one of the rarest central nervous system neoplasms compromising 0.25-0.5% of all primary brain tumors which was first described in the 1980s by Hassoun et al. and graded as WHO grade II [1, 2]. Ependymoma, astrocytoma, intraventricular oligodendroglioma, or primary cerebral neuroblastoma are differentials, but with immunohistochemistry, the definitive diagnosis can be made [3].

Young females are more prone to have CN than male [1, 4]. However, nowadays, it is believed that both sexes are equally affected. Commonly, patients present with severe headache vomiting and gait disturbance. On the other hand, some rare cases have severe symptom presentation, such as hemiparesis, seizures, and/or hemorrhage [4, 5].

No specific risk factors or geographic distribution were identified or reported except for a genetic component found in people of Asian descent than of other ethnic groups causing a larger proportion of CN to form [6].

Surgical resection is the treatment of choice having a good prognosis with complete resection with different approaches such as transcallosal, transcortical, and endoscopic. An adjuvant treatment is indicated in cases where complete resection is not possible such as radiation therapy [5, 6]. This is a case report of a CN with rare severe presentation and radiological findings in a 17-year-old male.

## 2. Case Presentation

**Figure 1:**
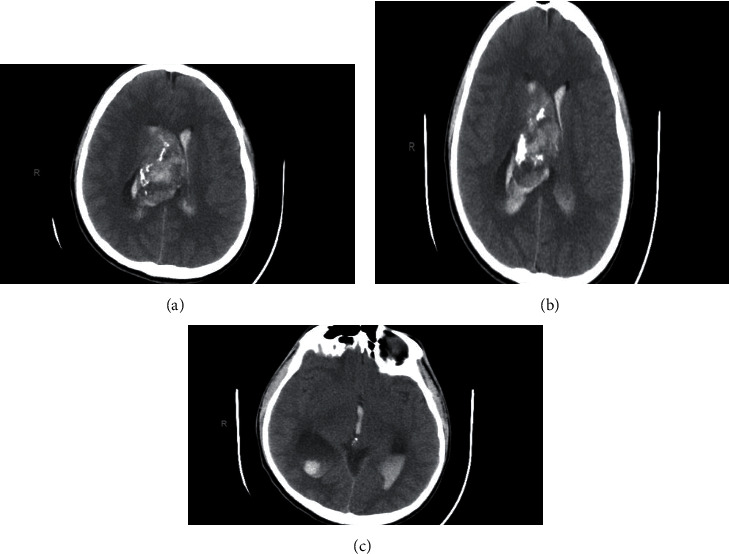
Head CT axial view without contrast.

**Figure 2:**
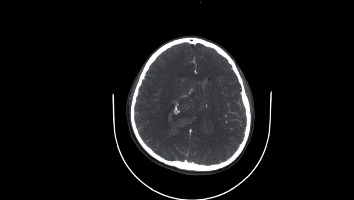
Head CT axial view with contrast.

This is a 17-year-old young man unknown to have any medical illness transferred to our center from other hospital as a case of hydrocephalus and intraventricular hemorrhage (IVH). He had a complain of headache for 3days that subsided with paracetamol. On the day of his presentation in the initial hospital, the patient suffered from severe headache with persistence vomiting and decreased level of consciousness with GCS of 11/15. However, the physical examination upon arrival to our center was GCS 9/15 and pupils equal reactive bilaterally with spontaneous movement for both upper and lower limbs. Consequently, head computer tomography (CT) was performed, and it showed a well-defined, lobulated large mass measuring 6 cm × 5 cm × 3 cm expanding the frontal horn and body of right lateral ventricle that was mainly hyperdense with heterogeneous attenuations and areas of calcifications (Figure 1). The lesion demonstrated no gross enhancement of the contrast (Figure 2). Also, it displaced the septum pellucidum and crossed the midline toward the left side about 3 cm. With mass effect on the frontal horn and body of left lateral ventricle, it obstructed the outflow of the ventricles resulting in obstructive hydrocephalus. The ventricular system was filled with blood which was shown as hyperdensities particularly seen in the dependent portions of occipital horns of bilateral lateral ventricles and third and fourth ventricles (Figure 1(c)).

**Figure 3:**
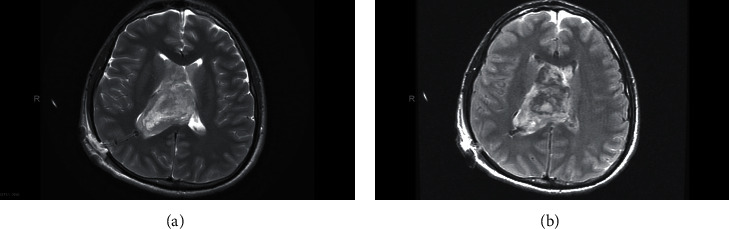
Head MRI axial view. (a) T2. (b) T1.

**Figure 4:**
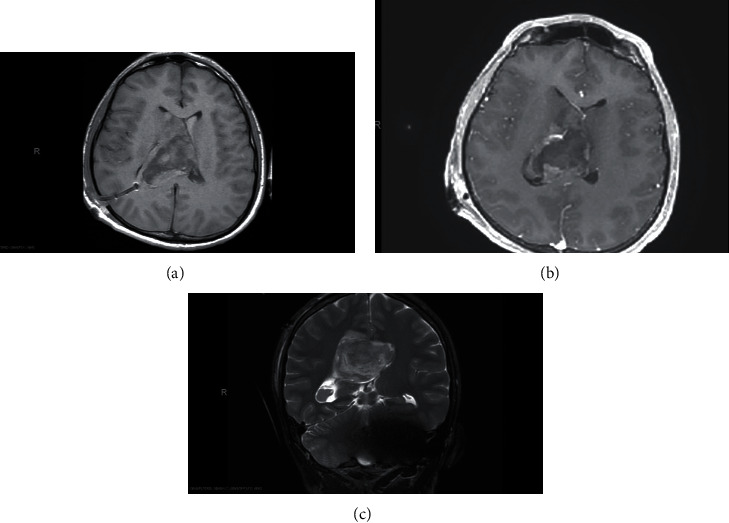
Brain MRI axial (a, b) and coronal view with contrast T2 (c).

Therefore, the patient underwent an emergency EVD insertion to relief intracranial pressure secondary to hydrocephalus. Bloody CSF came out under high pressure. Thereafter, a preoperative MRI of the brain was done, and it showed a heterogeneous ventricular mass about 40 mm in diameter on the right lateral ventricle which is hypointense signal on both T1and T2 with no contrast enhancement (Figure 3 and 4) suggestive of intraventricular tumor associated with intraventricular hemorrhage mainly in the right lateral ventricle. The preoperative differential diagnosis included ependymoma, subependymal giant cell astrocytoma, and central neurocytoma.

Accordingly, the patient underwent craniotomy for subtotal tumor resection via right transcortical approach. Using the navigation system, the cortex was opened at right frontal site exactly above the mass. The white matter was dissected posteriorly and medially reaching right frontal horn. While opening the right frontal horn, multiple clots were identified and removed until the ependymal wall was recognized. An abnormal gray mass was seen and debulked; a sample was taken for histopathology. Further dissection was carried out anteriorly and medially until there was no obvious abnormal tissue. Moreover, the ventricular system was investigated using endoscope medially anteriorly and posteriorly to confirm a good resection. A ventricular catheter was left in the ventricle.

Postoperatively, the patient was kept intubated and sedated for 10 days in the ICU. Postoperative examination while the patient was sedated revealed free movement of the limbs except for the left upper and lower limbs which showed motor power 1/5 and 2/5, respectively.

**Figure 5:**
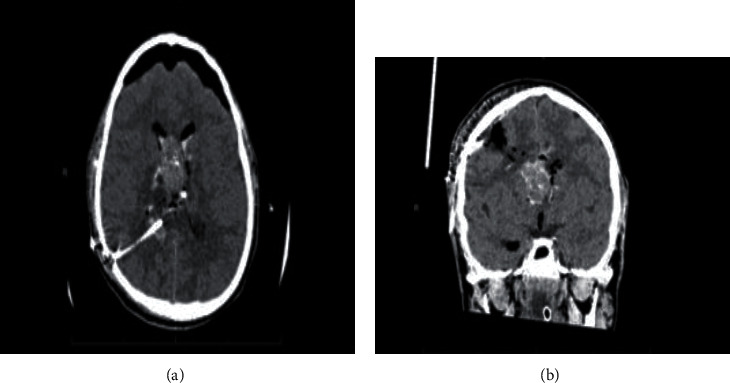
Head CT axial view post operatively.

**Figure 6:**
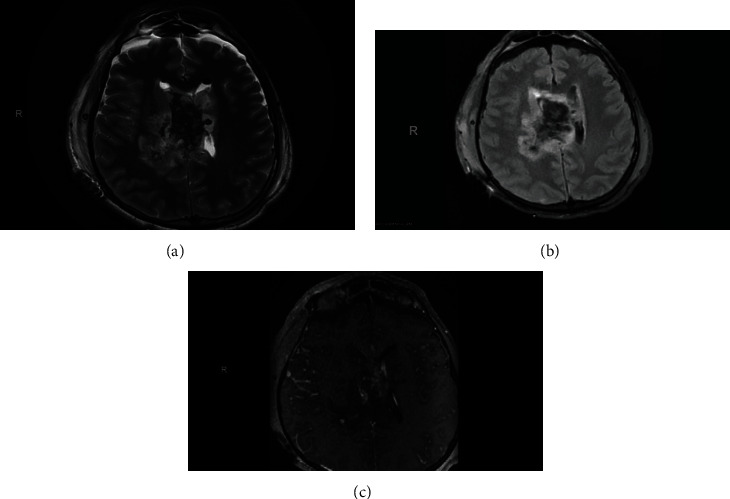
Brain MRI axial view T2 T1 postop.

After being whined off sedation, the patient was extubated and transferred to regular ward with his GCS improving. Later on, the patient started to follow orders and speak. However, he was returned to the operative room and underwent VP shunt insertion due to unresolving hydrocephalus and residual neoplasm (Figures 5 and 6). Afterward, due to the left hemiparesis, the patient started daily physiotherapy sessions to help him to regain his motor power in his left upper and lower limbs which were 3/5 and 4/5, respectively. Upon his discharge from the hospital, he started to mobilize with assistance.

**Figure 7:**
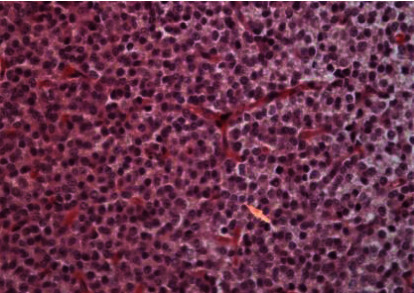
Hex400; monotonous bland neurocytoma cells.

**Figure 8:**
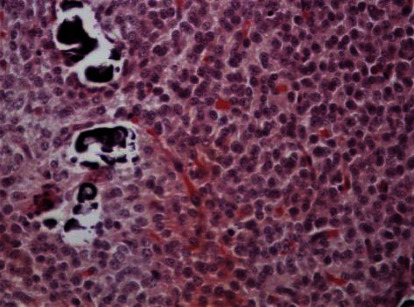
Calcification in neurocytoma.

**Figure 9:**
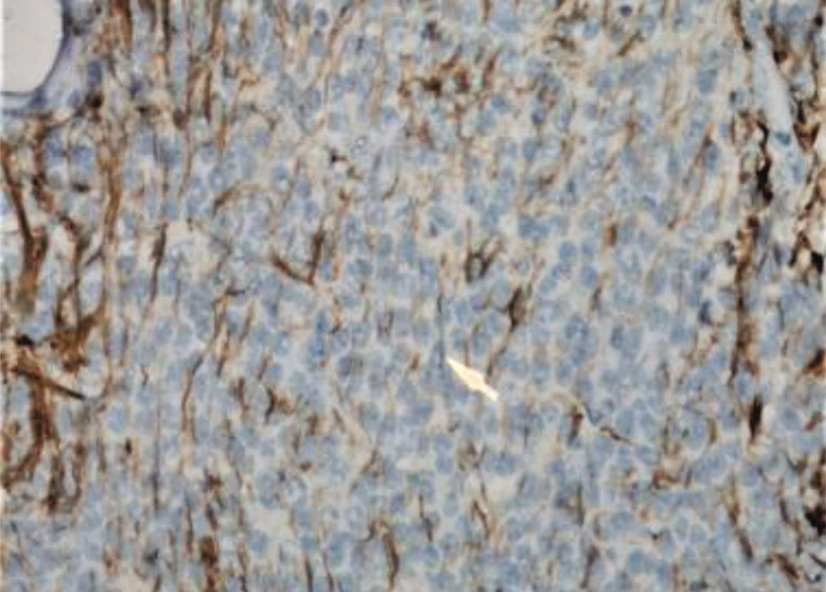
GFAP-negative neurocytoma cell expression seen in neuropil only.

**Figure 10:**
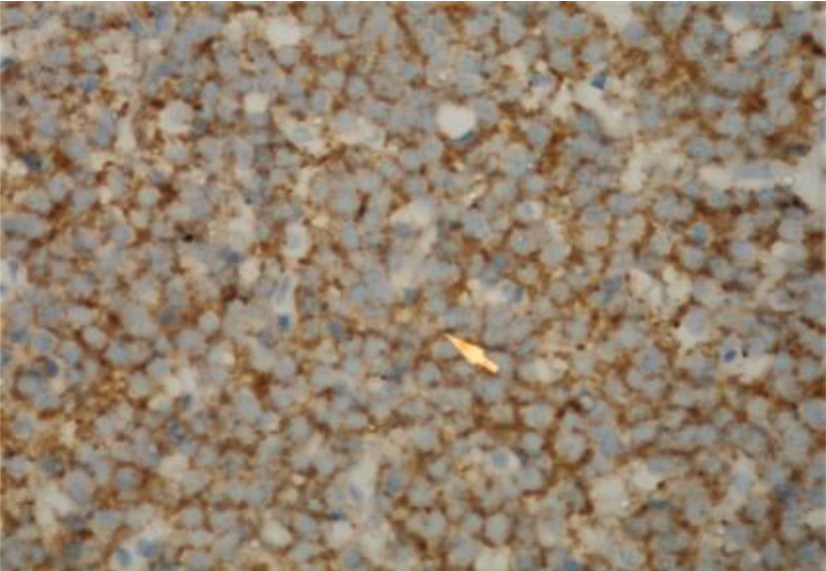
Very low ki67 labelling index.

Microscopically, the tumor was characterized by neuroepithelial tissue which was comprising of monotonous bland appearing cells showing fine chromatic nuclei and eosinophilic to clear modest cytoplasm, in a background of fibrillary matrix (Figure 7). These cells were seen in sheets and display a clear interface with neuropil. Ventricular lining of epithelial cells with intraventricular tumor was present in one focus. Many calcified bodies were observed (Figure 8). However, no necrosis or mitotic activity was noted. Well-controlled immunohistochemistry revealed diffuse expression of synaptophysin in tumor cells, which are negative for GFAP (Figure 9). Ki67 labeling index is <2% (Figure 10). The final pathological diagnosis was central neurocytoma (WHO grade II).

## 3. Discussion

Central neurocytoma is a rare well-differentiated neoplasm with ectodermal neuronal origin [7, 8]. It accounts for less than 1% of all primary tumors in central nervous system (CNS) with presentation of spontaneous intraventricular hemorrhage (IVH) is exquisitely rare [9, 10]. The tumor predominately occurs in young adults in between 20 and 40 years of age with peak in the third decade of life [9–13]. Most of the reports demonstrated no gender predilection [13, 14]. However, some studies showed slight male predominance [9, 10] with one retrospective study showed female predominance [11].

CN is typically deeply seated in midline structures, most commonly in the frontal horn of the lateral ventricle adjacent to foramen of Monro and attached to septum pellucidum which might extend to the third ventricle [11, 13, 15]. Nevertheless, there were some cases which reported other locations of neurocytoma, such as isolated neurocytoma in the fourth and third ventricles [14, 16], and extraventricular neurocytoma in spinal cord and brain parenchyma [11, 13, 14]. CN tends to be benign tumor though malignant variants were reported [7, 14]. Thereby, it may show favorable prognosis with adequate treatment. However, in some circumstances, it might exhibit aggressive behavior, especially with atypical variants [11].

### 3.1. Clinical Presentation

The initial clinical presentations of CN usually develop gradually within duration which varies from several hours to several years, but generally advance from 3 to 6 months [14, 17]. The most frequent presentations are headache, nausea, vomiting, dizziness, ataxia, papilledema, and visual disturbance. Most of these manifestations are consequences of raised intracranial pressure secondary to obstructive hydrocephalus [12, 15, 17, 18]. Ataxia and papilledema were reported as the most commonly presenting signs [14, 18] while headache was the most presenting symptom [15]. Other unusual presentations of CN include epilepsy, change in mental status, memory disturbance, aphasia, tinnitus, limb numbness, and weakness [13, 14, 17]. Rarely, CN acutely presents secondary to intraventricular or intertemporal hemorrhage [9, 17, 19].

Hemorrhage is not typical feature to occur in CN [12, 17, 19, 20]. Based on a recent retrospective study, 5 out of 69 (7.9%) patients with CN found to have hemorrhage. Also, Gunawat et al. has reported 2 cases of CN with IVH. Both cases presented with acute onset of headache and vomiting [19]. The definitive cause of the hemorrhage is unknown [9, 20], but several theories have been postulated; hypertensive heart disease, thrombocytopenia, thin wall tumor's vessels, and aneurysm on feeding vessels were proposed as sources of hemorrhage [9, 19, 20]. In fact, intratumoral hemorrhage is a useful indicator to differentiate CN from other intraventricular tumors which are less likely to bleed, the patient in this case report presented with symptoms of hydrocephalus and IVH with 3 days onset of severe headache accompanied by nausea, vomiting, and decrease level of consciousness [20].

### 3.2. Radiology

Radiologically, CN generally appears as well-demarcated lobulated mass in the lateral ventricle [16]. Its attachment to septum pellucidum is a characteristic feature of the tumor [20]. On CT scan, it either appears as mixed density or as isohyperdensity mass hypodense areas give heterogenous appearance of the tumor which correspond to cystic degeneration while patchy, coarse, clumped, and globular appearance indicate calcification which occurs in 25% to 50% of the cases [12, 13, 16, 20]. Infrequently, the tumor presents with hemorrhagic changes within it [9]. On contract enhancement CT, the tumor shows mild to moderate enhancement [9, 13, 15, 16].

Moreover, CN usually presents as heterogenous isointense to hypointense mass in T1-weighted MRI while isointense to hyperintense with soap-bubble multicystic appearance in T2-weighted MRI [13, 15, 16]. The contrast enhancement in MRI is variable, but moderate enhancement is frequently observed. When T1- and T2-weighted MRI hypointensities or patches without signals are seen, they indicate the presence of cyst, calcification, or hemorrhage [10, 13, 16, 17, 20, 21].

These neuroradiological characteristics were clearly observed in our patient and thus supporting the diagnosis of CN. The tumor appeared on CT as a well-defined hyperdense mass with heterogenous areas because of calcification which extended from the anterior portion of the right lateral ventricle with no contrast enhancement. Also, hyperdensities which filled the dependent portions of the ventricular system were observed indicating presence of IVH (Figures 1 and 2). On MRI, CN showed T1- and T2-weighted MRI heterogenous mass with hypointensity in both (Figures 3 and 4). Unfortunately, there are no established criteria that differentiae CN from other intraventricular tumors; hence, when CT scan or MRI are performed alone, CN might be misdiagnosed as oligodendrogliomas or ependymomas [13, 16].

Based on the radiological findings and taking the lesion exact location and the patients age into consideration, the preoperative differential diagnosis includes ependymoma, subependymal giant cell astrocytoma, central neurocytoma, and to a lesser extent oligodendroglioma, primary cerebral neuroblastoma, and choroid plexus papilloma. However, ependymoma and astrocytoma are characterized by the lack of intratumoral cysts and rarely have calcifications. Unlikely, intraventricular oligodendroglioma which is usually located within the body of the lateral ventricle can present with large intratumoral calcifications. Choroid plexus papilloma, on the other hand, is more common in supratentorial compartment in children and the posterior fossa in adults. It is located in the fourth ventricles in almost 70% of adults and the lateral ventricles in pediatrics. Unlike most other brain tumors, CNs are typically located in the supratentorial ventricular system extending to the third ventricle in 26% cases and rarely have extraventricular extensions [22]. It is noteworthy to mention that CN presented with spontaneous hemorrhage is superbly rare [23]. Such in our patient, the tumor presented with IVH contained in the lateral ventricles, and it is best seen in the posterior horns (Figure 1).

### 3.3. Surgery

The mainstay treatment of CN is a total resection which can be performed directly via transcortical, transcallosal, or via endoscopic neurosurgery [24, 25]. Total resection was achieved only in 30-50% of the cases [2, 8, 13] probably due to the tumor vascularity and its adherence to nearby structures [2]. Moreover, total resection of CN was associated with 99% 5-year survival rate in compassion to subtotal resection which was associated 86% 5-year survival rate [8, 13]. However, the serious risk of posing neurological deterioration may result from aggressive and excessive tumor resection; hence, a balance of advantages and disadvantages of the surgery should be considered [21]. Based on a systematic review, the complications rate after gross total resection of CN was 31.2%. In contrast, maximum safe resection with radiotherapy complication rate was 24% [25]. Correspondingly, the main goals of the surgery are to accomplish maximum resection of the tumor with minimum neurological sequel, to provide a sample for definitive histopathological diagnosis and to reestablish the CSF flow [14].

The choice of surgical approach is largely dependent on the surgeon experience and personal practice and tumor location [2, 15]. Yet, the surgical approach should be individualized [9, 24]. In general, surgical resection of CN carries challenge due to its huge size, location in the deep midline close to critical intraventricular structures, and occasional hypervascularity of the tumor [21]. Therefore, the transcallosal approach is mainly performed to operate in a tumor in the third ventricle or both lateral ventricles with normal ventricular size and to avoid cortical incision [2, 10, 15, 21, 25]. On the other hand, transcortical approach is used for easy access to the lateral ventricle tumor, to operate on large tumors, to reduce the risk of damaging the fornix and parasagittal vein, and to avoid making incision in the corpus callosum [2, 8, 15, 25]. With these advantages and disadvantages for each approach, complication should be taken into consideration; before choosing the approach, hemiparesis, memory loss, aphasia, mutism, and disconnection syndromes are the most reported complications associated with the transcallosal approach. In comparison, memory loss, seizures, confusion, hemiparesis, aphasia, and mutism are associated with transcortical approach complications [2]. Nevertheless, based on a retrospective analysis on 63 patients, no significant differences were reported between these surgical approaches in terms of extent of resection and neurological complications [15, 25]. Both of these surgical approaches carry similar major complications rates [2].

In fact, avoidance of open resection surgery complications has prompted the usage of endoscopic tumor removal surgery [25]. Cheng et al. found that endoscopic neurosurgery carries a relatively lower mortality and morbidity rates in comparison to classical open surgery to remove intraventricular tumor [24]. Because classical open surgery may result in subtotal resection owing to limited visualization and rich vascularization, endoscopic tumor resection was done with radiological confirmation for complete tumor removal resection in 3 patients with no significant complications [24]. Indeed, the hemorrhagic sequel from neuroendoscopic procedure of intraventricular tumor is 3.5% according to a retrospective study which was done on 86 patients [24].

For accuracy and safety's purposes, neuronavigation system was deployed as image-guided for neurosurgical operations for localization and delineation of brain lesions, therefore, ensuing maximum safe resection. However, intraoperative brain shift can limit its accuracy [26]. Thus, intraoperative ultrasound was implanted as fast and a real-time image which offers a correlation between the images obtained intraoperatively and preoperative images which are embedded in neuronavigation system [26, 27]. It also serves for identification unexpected intraoperative challenges, such as deliquoration of cerebrospinal fluid, lack of distinct microscopic boundaries, immediate visualization of hematoma, and detection of blood clot and tumor remnants in surgical cavities including the intraventricular chambers [27].

Besides, adjunctive radiotherapies have been used to treat recurrent and residual tumor [14, 28]. Mahavadi et al. has reported the recurrence rate after gross total resection was 23.9%, whereas 6.9% was the recurrence rate after gross total resection with radiotherapy (GTR+RT). However, GTR+RT may add morbidity while overall survival does not seem to be changed [25]. Adjunctive radiotherapies mitigate the risk of tumor progression after incomplete tumor resection and improve patient overall survival rate with maximum safe resection, which is 93.2% in al level comparable to GTR and GTR+RT (95.5% and 95.3%, respectively) [25]. Alternatively, a stereotactic radiosurgery is emergingly used as an accurate and safe option in comparison to conventional radiotherapy in case or tumor recurrence [29]. A meta-analysis study conducted on 150 patients who underwent radiosurgery showed the overall survival rate was 98% with follow-up period range from 3 to 149 months. About 25 of these patients were treated primarily by radiosurgery while the rest used it as adjunctive therapy. The study concluded that stereotactic radiosurgery is effective primary management for tumors that are less surgically amenable [23]. Likewise, a systematic review demonstrated consistent findings [25].

In our patient, due to the large tetraventricular hemorrhage which played role in the onset of the postoperative hydrocephalus, the transcallosal route was unsuitable; therefore, transcortical approach was favored over transcallosal approach in our case, a subtotal tumor resection was accomplished via transcortical approach because of the high vascularity of the tumor. Postoperatively, the patient was managed accordingly, but no radiotherapy was performed. In addition, the only documented complications were hemiparesis grade 3 and unresolving hydrocephalus.

### 3.4. Histological

**Figure 11:**
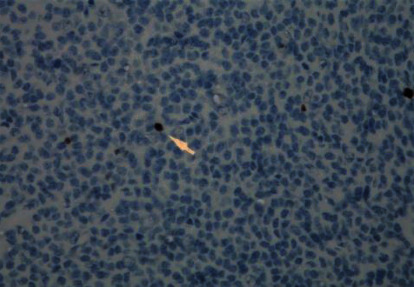
IHCx400; synaptophysin expression in neurocytoma.

The histopathological diagnosis of CN is challenging as many of its histopathological features resemble other neoplasms of CNS, thus resulting in misdiagnosis. Therefore, the definitive diagnosis of CN relies mainly on immunohistochemistry to determine the neuronal origin of the tumor. The most reliable diagnostic marker in immunohistochemistry is antibodies to synaptophysin which labels CN in both fibrillary and perivascular areas. Furthermore, positive neuron-specific enolase and negative for glial fibrillary acidic protein can be used as other features of immunohistochemistry for CN [13, 14]. In this case report, the immunohistochemistry shows diffuse expression of synaptophysin in tumor cells (Figure 11), which are negative for GFAP (Figure 9) with Ki67 labeling index is <2% (Figure 10). Generally, the histological features of CN show uniform cells with round or oval nuclei and loose fibrillary matrix background [9]. These features are consistent with our findings, hence supporting the diagnosis of CN.

## 4. Conclusion

CN is a slow-growing benign neoplasm of the central nervous system that has an excellent prognosis. Although the best long-term survival rates and local tumor control correlates with total resection which is the most preferable management, adjuvant radiotherapy is considered following subtotal resection with residual CN, large-sized CN, or deep-seated CNs.

We describe here a rare case of central neurocytoma with atypical presentation and radiological finding; however, central neurocytoma should be considered as a differential diagnosis in slow growing neoplasms with hemorrhagic extension to the ventricles; a total safe resection should be carried out; if it cannot be achieved, a subtotal resection followed by adjuvant radiotherapy should be considered.
